# PU.1 and IRF8 Modulate Activation of NLRP3 Inflammasome *via* Regulating Its Expression in Human Macrophages

**DOI:** 10.3389/fimmu.2021.649572

**Published:** 2021-04-07

**Authors:** Takuya Yashiro, Machiko Yamamoto, Sanae Araumi, Mutsuko Hara, Kyoko Yogo, Koichiro Uchida, Kazumi Kasakura, Chiharu Nishiyama

**Affiliations:** ^1^Department of Biological Science and Technology, Faculty of Industrial Science and Technology, Tokyo University of Science, Katsushika-Ku, Japan; ^2^Atopy (Allergy) Research Center, Juntendo University Graduate School of Medicine, Bunkyo-ku, Japan; ^3^Juntendo University Advanced Research Institute for Health Science, Bunkyo-ku, Japan

**Keywords:** N LRP3, inflammasome, PU.1, IRF8, macrophage

## Abstract

NLRP3 inflammasomes play crucial roles in the initiation of host defense by converting pro-Caspase-1 to mature Caspase-1, which in turn processes immature IL-1β and IL-18 into their biologically active forms. Although NLRP3 expression is restricted to monocytic lineages such as monocytes, macrophages, and dendritic cells, the mechanisms determining the lineage-specific expression of NLRP3 remain largely unknown. In this study, we investigated the transcription factors involved in cell-type-specific transcription of *NLRP3*. We found that a distal, rather than a proximal, promoter of human *NLRP3* was predominantly used in the human monocytic cell lines and macrophages. Reporter analysis showed that an Ets/IRF composite element (EICE) at -309/-300 and an Ets motif at +5/+8 were critical for transcriptional activity of the distal promoter. Electrophoretic mobility shift assays and chromatin immunoprecipitation assays demonstrated that two transcription factors, PU.1 and IRF8, both of which play essential roles in development and gene expression of the monocytic lineage, were bound to the EICE site, whereas PU.1 alone was bound to the Ets site. Knockdown of PU.1 and/or IRF8 mediated by small interfering RNA downregulated expression of NLRP3 and related molecules and markedly diminished the LPS-induced release of IL-1β in THP-1, suggesting that activity of the NLRP3 inflammasome was suppressed by knockdown of PU.1 and IRF8. Taken together, these results indicate that PU.1 and IRF8 are involved in the monocytic lineage-specific expression of NLRP3 by binding to regulatory elements within its promoter and that PU.1 and IRF8 are potential targets for regulating the activity of the NLRP3 inflammasome.

## Introduction

Nod-like receptor pyrin domain containing 3 (NLRP3) is a pattern-recognition receptor belonging to the NLR family and is mainly expressed in monocytes, macrophages, and dendritic cells (DCs). After interacting with pathogen-associated molecular patterns or damage-associated molecular patterns, NLRP3 forms the NLRP3 inflammasome complex with pro-Caspase-1 in the presence of the adapter protein apoptotic scaffold protein containing a caspase recruitment domain (ASC), and subsequently converts pro-Caspase-1 to Caspase-1 (1). Activated Caspase-1 cleaves pro-IL-1β and pro-IL-18 into their biologically active forms (2, 3). Proper activation of the NLRP3 inflammasome plays an important role in host defense (4–6). In contrast, chronic activation of the NLRP3 inflammasome is involved in the onset and progression of various diseases such as auto-inflammatory diseases, autoimmune diseases, diabetes, arteriosclerosis, cancer, and allergic diseases (7–10).

The Ets family transcription factor PU.1, encoded by *Spi1*, is essential for the development of myeloid and lymphoid lineages from hematopoietic stem cells (11). Mice deficient in PU.1 die either in late gestation or shortly after birth because of severe impairment of hematopoiesis (12, 13). During lineage commitment, PU.1 regulates the gene expression of cytokine receptors such as M-CSFR, G-CSFR, GM-CSFRα, and IL-7Rα, which are essential for the development of monocytes, granulocytes, and lymphocytes (11, 14). In addition, PU.1 is abundantly expressed even after terminal differentiation, particularly in macrophages and DCs, and we found that PU.1 transactivates genes encoding molecules with key roles in DCs, such as CIITA (15–20). These findings highlight the crucial roles of PU.1 in innate and adaptive immunity. Although PU.1 can bind as a monomer to the Ets motif [(G/A)GAA] within the regulatory region of its target genes, PU.1 can also bind to the Ets-IRF composite element (EICE) [GAAANN(G/A)GAA] by forming a heterodimer with interferon regulatory factor 4 (IRF4) or IRF8 (21). A series of studies demonstrated that IRF4 and IRF8 play essential roles in the development and phenotype of monocytes, macrophages, and DCs (22, 23). In DCs, IRF8 and IRF4 play important roles by determining the commitment of conventional type 1 dendritic cell (cDC1) and conventional type 2 dendritic cell (cDC2), respectively (24–26). *Irf8*^-/-^ mice exhibit immunodeficiency and chronic myeloid leukemia-like disease because of impaired macrophage development and expansion of granulocytes (27). PU.1 and IRF8 cooperatively regulate macrophage-specific genes such as cystatin C and cathepsin C (28).

Several nuclear molecules have been identified as transcriptional regulators of *NLRP3*. NF-κB and pregnane X receptor (PXR) positively regulate gene expression of mouse and human *NLRP3*, respectively, by directly binding to the promoter region (29, 30). In contrast, aryl hydrocarbon receptor (AhR) and growth factor independence 1 (GFI1) suppress *mNlrp3* transcription by inhibiting NF-κB activity (31, 32). Recently, it was reported that IRF8 in cDC1 and IRF4 in cDC2 suppress activation of the NLRP3 inflammasome by negatively regulating expression of *mNlrp3*, whereas macrophages, in which the expression levels of IRF8 and IRF4 are apparently lower than those in cDC1 and cDC2, respectively, express NLRP3 and exhibit NLRP3 inflammasome activity (33). However, the hematopoietic cell-specific transcription factors transactivating *NLRP3* in human macrophages and the role of PU.1 in determining the activity of the NLRP3 inflammasome in macrophages are largely unknown.

In the present study, we investigated the gene expression mechanism of *hNLRP3* by using a human monocytic cell lines and macrophages, and found that PU.1 and IRF8 positively regulate *hNLRP3* expression by binding to the EICE motif within its promoter region.

## Materials and Methods

### Cell Culture

THP-1 and U937 cells (ATCC, Manassas, VA, USA) were cultured in RPMI1640 supplemented with 10% fetal calf serum, 100 U/mL penicillin, and 100 mg/mL streptomycin at 37°C in a humidified atmosphere in the presence of 5% CO_2_. Bone marrow-derived macrophages (BMDMs) were generated as previously described (19). Human macrophages were generated by culturing CD14^+^ monocytes isolated from the authors’ (TY and MH) peripheral blood mononuclear cells in the presence of 50 ng/mL hM-CSF (BioLegend, San Diego, CA, USA) for 5 or 6 days. Human and animal experiments were performed in accordance with the approved guidelines of the Institutional Review Board of Tokyo University of Science (Tokyo, Japan).

### Reverse Transcription-PCR and Quantitative PCR

RNA was extracted and reverse transcription (RT) performed as previously described (20). RT-PCR was conducted using KOD FX (TOYOBO, Osaka, Japan) and the following primers: forward1 5′-CTAGCTGTTCCTGAGGCTGG-3′, forward2 5′-GCCTTCAGTTTGGAGGAACTG-3′, and reverse 5′-GAAGATCCACACGGCCATGG-3′. Quantitative PCR was performed as previously described (34). The TaqMan Gene Expression Assays (Applied Biosystems, Foster City, CA, USA) and sequences of synthesized oligonucleotide primers are listed in **Supplementary Table 1**.

### Luciferase Assay

The distal and proximal promoters of *NLRP3* were amplified from human genomic DNA by PCR and inserted into the multi-cloning site of pGL-4.10 (Promega, Madison, WI, USA) to generate reporter plasmids. Mutant reporter plasmids were generated using a PrimeSTAR Mutagenesis basal kit (TaKaRa Bio, Shiga, Japan). The nucleotide sequences of the primers are listed in **Supplementary Table 2**. THP-1 cells were transfected with 400 ng reporter plasmid and 600 ng pRL-CMV (Promega) using FuGENE HD (Promega). Luciferase activity was determined at 48 h after transfection using an ARVO X Light (PerkinElmer, Waltham, MA, USA) plate reader and Dual-Luciferase assay kit (Promega).

### Electrophoresis Mobility Shift Assay

The electrophoretic mobility shift assay (EMSA) was performed based on a method described previously (35). Fluorescein-labeled or non-labeled double-stranded oligonucleotides of the target sequence were prepared as probes and competitors, respectively. Nuclear proteins were extracted from THP-1 cells as previously described (36). Anti-PU.1 antibody (D19), anti-IRF4 antibody (M17), and anti-IRF8 (ICSBP) antibody (C19) (all from Santa Cruz Biotechnology, Dallas, TX, USA) were used for the supershift assays. The band shifts on a polyacrylamide gel were analyzed with a Typhoon FLA 9500 laser scanner (GE Healthcare, Chicago, IL, USA).

### Chromatin Immunoprecipitation Assay

Chromatin immunoprecipitation (ChIP) assays were performed as previously described (34, 37). Anti-PU.1 antibody (D19), anti-IRF8 antibody (C19), and goat IgG (no. 02-6202; Invitrogen, Carlsbad, CA, USA) were used. The amount of precipitated DNA was determined by quantitative PCR using an Applied Biosystems Step-One Real-time PCR system. The nucleotide sequences of the PCR primer sets were as follows: -337/-275, forward, 5′- TTTACTCACTCGCATGGCATGT-3′, reverse, 5′- CTGCAACGGCTCCACTGA-3′; negative control, forward, 5′- GAGGAGTAGATAGGCAGGAATGGA-3′, reverse, 5′- AATGTCAAGATGCCTCAGACTCACT-3′.

### ChIP-Seq Data Analysis

ChIP-seq data of human macrophages with an anti-H3K27ac antibody (GSM2942925 and GSM2942926) were obtained from Gene Expression Omnibus (38). The data were analyzed by using Integrative Genomics Viewer (IGV).

### Small Interfering RNA Transfection

*SPI1* siRNA (HSS186060), *IRF8* siRNA (HSS105171), *Spi1* siRNA (MSS247676), *Irf8* siRNA (MSS236848), and a Stealth RNAi siRNA negative control set were purchased from Thermo Fisher Scientific (Waltham, MA, USA). THP-1 cells (2 × 10^6^ cells) suspended in R buffer were mixed with 200 pmol siRNA and then transfected with the Neon transfection system (Thermo Fisher Scientific) setting program No.5. Transfection into BMDMs and human macrophages was performed with Nucleofector 2b (Lonza, Basel, Switzerland) using the Amaxa Mouse Dendritic Cell Nucleofector Kit (Lonza), as previously described (18).

### Western Blotting

Western blotting was performed as previously described (39). Anti-NLRP3 antibody (D4D8T, Cell Signaling Technology, Danvers, MA, USA), anti-Caspase-1 antibody (EPR19672, Abcam, Cambridge, UK), anti-ASC antibody (B-3, Santa Cruz Biotechnology), anti-PU.1 antibody (T21, Santa Cruz Biotechnology), anti-IRF8 antibody (C19), and anti-β-actin antibody (AC-15, Sigma-Aldrich, St. Louis, MO, USA) were used as primary antibodies.

### Measurement of IL-1β Protein Level

The concentration of cytokines in the culture medium was determined using enzyme-linked immunosorbent assay (ELISA) kits for IL-1β (BioLegend) following the manufacturer’s instructions.

### Detection of Dead Cells

Dead cells were stained with 1 μg/mL DAPI (Nacalai Tesque, Kyoto, Japan) and analyzed with a MACSQuant flow cytometer (Miltenyi Biotech, Gladbach Bergisch, Germany).

### Statistical Analysis

Multiple groups were compared by one-way ANOVA and Tukey–Kramer test. The difference between any two groups was analyzed by unpaired student’s *t*-test. *p* < 0.05 was considered as statistically significant.

## Results

### Distal Promoter of hNLRP3 Predominates in Human Monocytes and Macrophages

**Figure 1:**
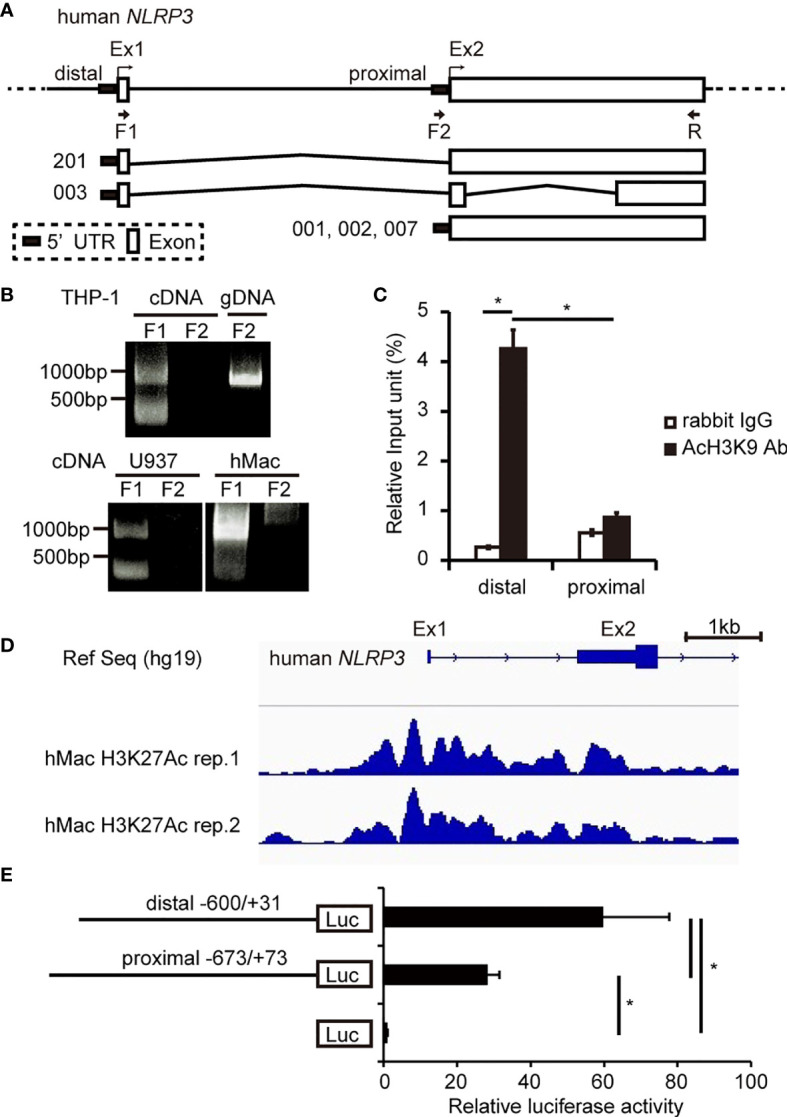
Determination of the predominant promoter for *hNLRP3* transcription in human monocytes and macrophages. **(A)** Human *NLRP3* structure. **(B)** RT-PCR using primers indicated in **(A)** and cDNA of THP-1, U937, or human macrophages (hMac) as a template. THP-1 genomic DNA was used as a positive control in PCR. **(C)** ChIP assays with THP-1 cells were performed using anti-AcH3K9 antibody or rabbit IgG. Co-immunoprecipitated DNA was quantified by qPCR with specific primer sets for the indicated region of *hNLRP3*. **(D)** ChIP-seq of hMac with an anti-acetyl histone H3K27 (H3K27Ac). The region around the distal promoter and the proximal promoter of hNLRP3 is shown. **(E)** THP-1 cells were transfected with the reporter vectors. Luciferase activities were determined at 48 h after transfection by normalizing firefly activities to *Renilla* luciferase activity. Data are expressed as the ratio to the empty vector. Data are presented as the mean + S.D. (*n* = 3). **(C, E)** *p < 0.05, Tukey–Kramer test.

Six protein-cording transcripts of *hNLRP3* are registered in the Ensembl Genome Browser (http://asia.ensembl.org/index.html). They are classified into two groups: one is transcribed from exon 1 (NLRP3-201, -003), and the other is transcribed from exon 2 (NLRP3-001, -002, -004, -007) (**Figure 1A**). In the current study, we named the 5′-flanking region of exon 1 as the distal promoter and that of exon 2 as the proximal promoter (**Figure 1A**). To determine which promoter is predominantly used in human monocytes and macrophages, we performed RT-PCR using their cDNA as a template and primers designed to amplify the transcript driven by the distal or proximal promoter. As shown in **Figure 1B**, the transcript from the distal promoter, which has a predicted size of 994 bp (NLRP3-201) or 352 bp (NLRP3-003), was detected in THP-1, U937, and human macrophages, suggesting that the distal promoter is active in monocytes and macrophages. In contrast, no PCR product was detected when we used the F2+R primer set to amplify the transcript driven by the proximal promoter (**Figure 1B**). This primer set was able to amplify DNA when THP-1 genomic DNA was used as a template, suggesting that the F2 primer is workable and that the proximal promoter is inactive in monocytes and macrophages. We next carried out a ChIP-qPCR assay using an anti-acetyl histone H3 K9 antibody to assess the state of chromatin modification. We found that histone H3 in the distal promoter was highly acetylated compared to that in the proximal promoter (**Figure 1C**), suggesting that the distal promoter predominates in THP-1 cells. Furthermore, we investigated the histone modification by analyzing ChIP-seq data uploaded to the Gene Expression Omnibus (https://www.ncbi.nlm.nih.gov/geo/) (38). Histone H3 K27 in the distal promoter was highly acetylated compared to that in the proximal promoter in human macrophages (**Figure 1D**). We then performed luciferase reporter assays using vectors containing each of the promoters. Luciferase activity derived from the distal promoter was significantly higher than that from the proximal promoter, supporting that the distal promoter is predominantly active in THP-1 cells (**Figure 1E**). Collectively, the results indicate that *hNLRP3* is predominantly transcribed from exon 1 using the distal promoter in human monocytes and macrophages.

### Identification of Cis-Acting Elements in the Human NLRP3 Promoter

**Figure 2:**
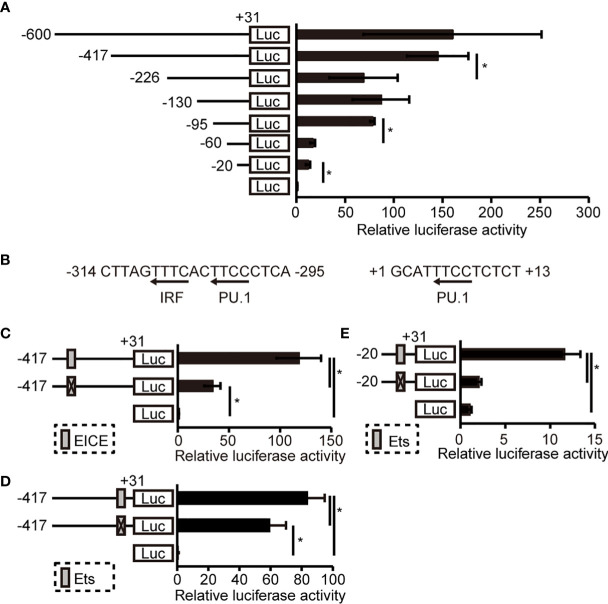
Determination of *cis* element(s) in the distal promoter of *hNLRP3* **(A, C–E)** THP-1 cells were transfected with reporter vectors containing various lengths of the distal promoter **(A)** or bearing mutations in the indicated sites **(C–E)**. Luciferase activities were determined at 48 h after transfection by normalizing firefly activities to *Renilla* luciferase activity. **(B)** PU.1- and IRF-binding sites within the *cis*-elements. Data are expressed as the ratio to the empty vector. Data are presented as the mean + S.D. (*n* = 3). *p < 0.05, two-tailed student’s *t*-test analysis.

To identify *cis*-acting element(s) in the distal promoter of *hNLRP3*, we performed luciferase reporter assays with vectors containing various lengths of the *hNLRP3* promoter. As shown in **Figure 2A**, luciferase activities were significantly decreased by deletion of -417/-227, -95/-61, and -20/+31, suggesting that these regions include *cis*-acting element(s). We then explored *cis*-acting elements within these regions using JASPAR (http://jaspar.genereg.net/), which is a database of transcription factor binding profiles. Although no promising sites recognizable by transcription factors were detected in -95/-61, we found a PU.1- and IRF-binding site at -309/-300 and PU.1-binding site at +5/+8 (**Figure 2B**). As both PU.1 and IRFs are known to be involved in monocyte-specific gene expression (28), we assessed whether these sites contribute to promoter activity by using a mutated version of the luciferase vectors. Promoter activity was significantly reduced by nucleotide replacement at -309/-300 from TTTCACTTCC into TTGTCGACCC and at +4/+9 from TTTCCT into GTCGAC (**Figures 2C, D**). Additionally, replacement at +4/+9 in the minimal promoter resulted in the loss of promoter activity (**Figure 2E**). The results indicate that these *cis*-elements are essential for the promoter activity of *hNLRP3*.

### Identification of Trans-Acting Factors in the Human NLRP3 Promoter

**Figure 3:**
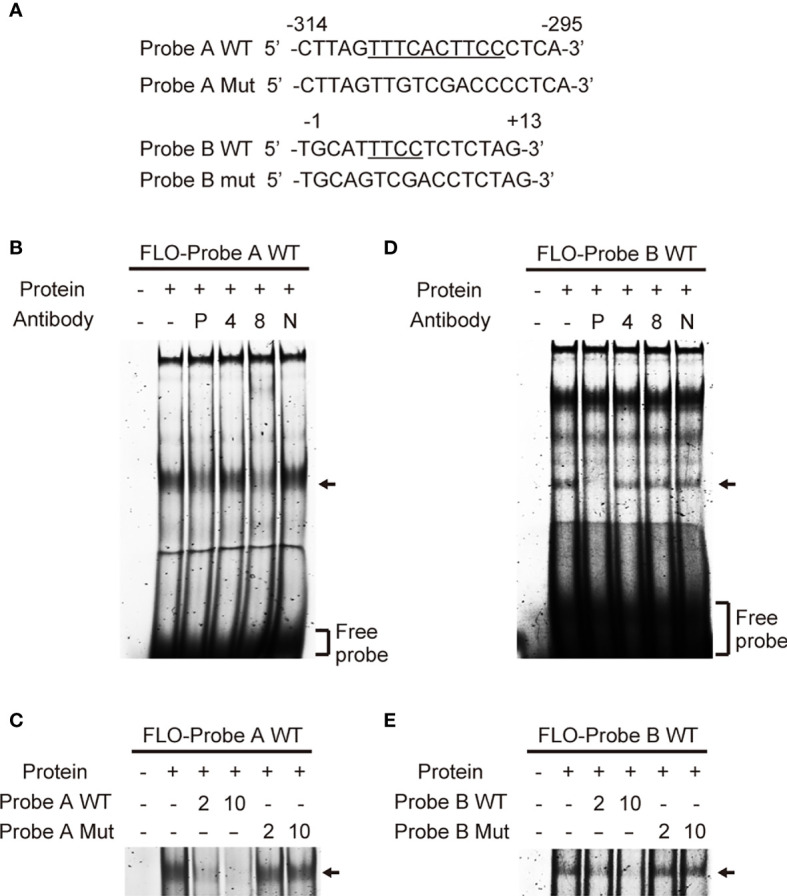
Identification of transcription factors that bind to the *cis*-elements. **(A)** Sequences of the probes used in electrophoretic mobility shift assay. **(B, D)** Fluorescein (FLO)-labeled probe A **(B)** or B **(D)** were incubated with nuclear extracts of THP-1 cells in the presence of anti-PU.1 (P), anti-IRF4 (4), anti-IRF8 (8) or nonspecific (N) antibodies. **(C, E)** FLO-labeled probe A **(C)** or B **(E)** were incubated with nuclear extracts of THP-1 cells in the presence of 2- or 10-fold amounts of unlabeled wild-type or mutated oligonucleotides. After electrophoresis on 5% acrylamide gels, fluorescence was detected.

To identify transcription factors that bind to the *cis*-elements we identified, we performed EMSA using THP-1 nuclear extracts and fluorescein (FLO)-labeled DNA probes (**Figure 3A**). Among the several bands visible in *lane 2* (**Figure 3B**), which contained a mixture of the nuclear extract and labeled probe A containing the EICE, the major band was diminished by addition of an anti-PU.1 antibody (**Figure 3B** *lane 3*). Addition of an anti-IRF4 antibody did not affect the band pattern but the intensity of the major band was reduced in the presence of an anti-IRF8 antibody (**Figure 3B** *lanes 4, 5*). Furthermore, this band gradually disappeared upon addition of an excess amount of non-labeled WT probe A (**Figure 3C** *lanes 3, 4*) but not upon addition of the non-labeled probe A mutant bearing a nucleotide replacement at the EICE (**Figure 3C** *lanes 5, 6*). These results suggest that PU.1 and IRF8, but not IRF4, can bind to the -309/-300 EICE by forming a heterodimer.

In an EMSA using probe B, among the several shifted bands observed in the lane (**Figure 3D** *lane 2*), in which a mixture of nuclear proteins and the probe was loaded, the shifted band showing the highest mobility (marked with an arrow) disappeared in the presence of the anti-PU.1 antibody. In contrast, addition of either the anti-IRF4 or the anti-IRF8 antibody did not affect the band intensity (**Figure 3B** *lanes 3–5*). As shown in **Figure 3E**, the mutant oligonucleotide lacking the Ets motif did not competitively inhibit the interaction between the wild-type probe and nuclear PU.1 protein. These results suggest that PU.1 binds directly to an Ets motif at +5/+8 in *NLRP3*.

### PU.1 and IRF8 Bind to the Proximal Region of Human NLRP3 Ppromoter

**Figure 4:**
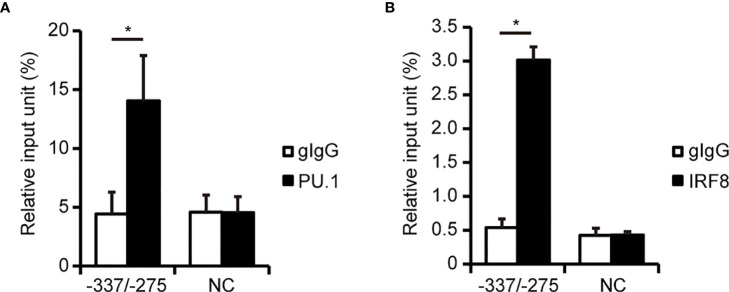
PU.1 and IRF8 bind to the *hNLRP3* promoter in THP-1 cells. **(A, B)** THP-1 cells were subjected to ChIP assays using anti-PU.1 antibody (PU.1) **(A)**, anti-IRF8 antibody **(B)**, or goat IgG (gIgG). Coimmunoprecipitated DNA was quantified by qPCR with primers amplifying the indicated regions of the *hNLRP3* promoter. Data are presented as the mean + S.D. (*n* = 3). *p < 0.05, two-tailed student’s *t*-test analysis.

To investigate whether PU.1 and IRF8 bind to the *hNLRP3* promoter in THP-1 cells, we performed a ChIP-qPCR assay. When we conducted quantitative PCR with specific primer sets amplifying around the EICE, the amount of immunoprecipitated chromosomal DNA with the anti-PU.1 antibody was significantly higher than that obtained with the isotype control (**Figure 4A**). However, there was no difference between the anti-PU.1 antibody and isotype control upon amplification of another region which includes neither EICE nor Ets (**Figure 4A**, NC). These results suggest that PU.1 specifically binds to the identified EICE in THP-1 cells. Furthermore, the ChIP-qPCR assay with the anti-IRF8 antibody and specific primer sets amplifying around the EICE clearly showed that IRF8 specifically binds to the identified EICE in THP-1 cells (**Figure 4B**).

### Both PU.1 and IRF8 Are Involved in Gene Expression of Human NLRP3

**Figure 5:**
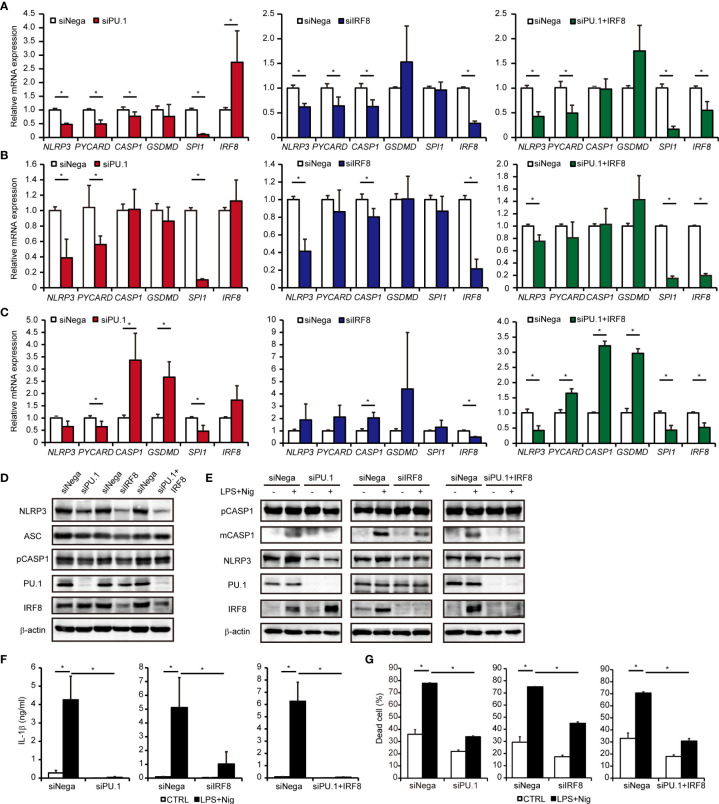
Effects of PU.1 and IRF8 knockdown on NLRP3 expression and activation of the NLRP3 inflammasome in human macrophages. **(A–D)** THP-1 **(A, D)**, U937 **(B)**, or human macrophages **(C)** were transfected with *SPI1* siRNA (siPU.1), *IRF8* siRNA (siIRF8), or negative control siRNA (siNega) and incubated for 48 h. **(A–C)** Relative mRNA expression was determined by qPCR and normalized to expression of *Gapdh* mRNA. **(D)** Cellular protein expression was determined by western blotting. **(E–G)** THP-1 cells were differentiated into macrophages by stimulating with 25 ng/mL PMA for 3 h and incubation for 21 h. Cells were primed with 1 μg/mL LPS for 4 h and then stimulated with 10 μM nigericin for 0.5 h **(E, F)** or 2 h **(G)**. **(E)** Cellular protein expression was determined by western blotting. **(F)** Amount of IL-1β in the culture medium was measured by ELISA. **(G)** Rate of dead cells was determined by flow cytometry. Data are presented as the mean + S.D. (*n* = 3). **(A–C)** *p < 0.05, two-tailed student’s *t*-test analysis. **(F, G)** *p < 0.05, Tukey–Kramer test.

To investigate whether PU.1 and IRF8 are involved in the gene expression of *hNLRP3*, we introduced *SPI1* siRNA and *IRF8* siRNA into two monocytic cell lines and quantified the mRNA levels of NLRP3 and related molecules by qPCR. Under PU.1 or IRF8 knockdown conditions, *hNLRP3* mRNA levels were significantly decreased in THP-1 and U937 cells (**Figures 5A, B**). In the case of PU.1 and IRF8 double knockdown, *hNLRP3* mRNA levels were significantly decreased but the reduction was not greater than that of the single knockdown (**Figures 5A, B**). To exclude off-target effects, we introduced another siRNA targeting other sequences of *SPI1* and *IRF8* into THP-1 cells and obtained similar results (**Supplementary Figure 1**). To examine the role of PU.1 and IRF8 on the expression of *hNLRP3* in primary cultured cells, we generated human macrophages by culturing CD14^+^ monocytes with M-CSF. Although single knockdown of PU.1 or IRF8 did not affect the mRNA levels of *hNLRP3*, double knockdown significantly decreased *hNLRP3* mRNA levels in human primary cultured macrophages (**Figure 5C**). We next examined protein levels by western blotting and found that NLRP3 protein levels were significantly decreased by single and double knockdown of PU.1 and IRF8 (**Figure 5D**). Based on these results, PU.1 and IRF8 cooperatively transactivate *hNLRP3*. There was no consistent change in these three cells regarding the effect of PU.1 and/or IRF8 knockdown on mRNA levels of *hPYCARD*, *hCASP1*, and *hGSDMD*, which are other components of the NLRP3 inflammasome (**Figures 5A–C**). Consistent with the mRNA levels, the protein levels of CASP1 and ASC were slightly decreased by single knockdown but not double knockdown (**Figure 5B**). Furthermore, we measured the expression levels of other NLRs family members, and found that *hNLRC4* mRNA expression was significantly decreased by PU.1 knockdown in THP-1, U937, and macrophages (**Supplementary Figure 2**). These results suggest that PU.1 and IRF8 control the function of several inflammasomes by regulating the expression of their components.

### Effect of PU.1 and IRF8 Knockdown on Inflammasome Activation

We examined whether knockdown of PU.1 and IRF8 leads to a functional defect in the NLRP3 inflammasome. As shown in **Figure 5E**, LPS and nigericin-induced maturation of CASP1 (40) was severely suppressed by PU.1 and/or IRF8 knockdown. In addition, the levels of mature IL-1β protein released from LPS-primed and nigericin-stimulated THP-1 cells were significantly decreased by PU.1 and/or IRF8 knockdown (**Figure 5F**). As NLRP3 inflammasome leads to pyroptotic cell death by activating pore-forming GSDMD (41, 42), we investigated the effect of knockdown of PU.1 and IRF8 on cell death. The cell death rate of THP-1 was markedly elevated by LPS and nigericin stimulation, but the elevation was attenuated by PU.1 and/or IRF8 knockdown (**Figure 5G**). These results suggest that downregulating NLRP3 expression by knockdown of PU.1 and IRF8 leads to inhibition of NLRP3 inflammasome activity.

### IRF8 Is Not Involved in NLRP3 Expression in Mouse Macrophages

**Figure 6:**
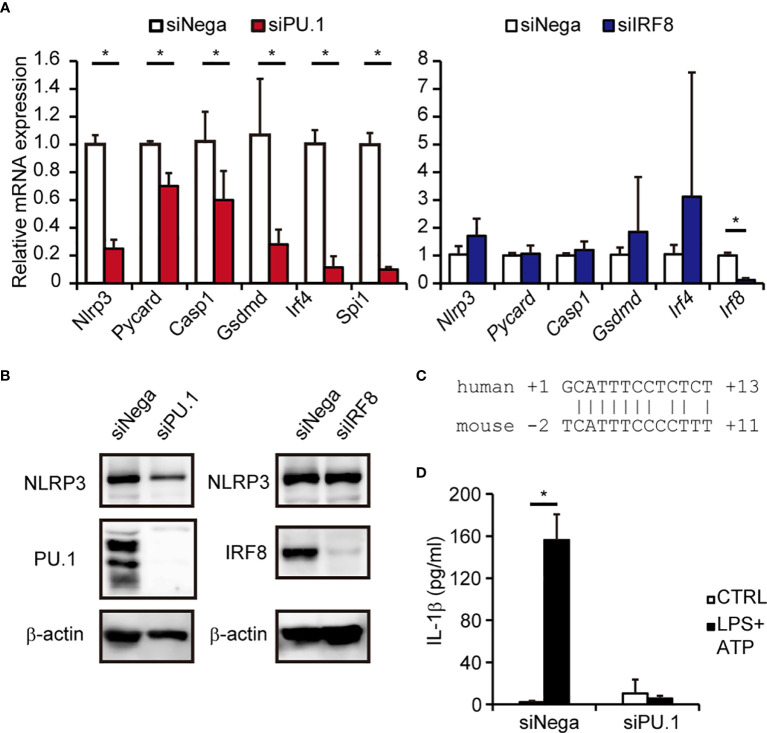
Effects of PU.1 and IRF8 knockdown on NLRP3 expression and activation of the NLRP3 inflammasome in mouse macrophages. **(A, B, D)** BMDMs were transfected with *Spi1* siRNA (siPU.1), *Irf8* siRNA (siIRF8), or negative control siRNA (siNega) and incubated for 48 h. **(A)** Relative mRNA expression was determined by qPCR and normalized to *Gapdh* mRNA expression. **(B)** Cellular protein expression was determined by western blotting. **(D)** Cells were primed with 1 μg/mL LPS for 4 h and then stimulated with 2 mM ATP for 0.5 h. The amount of IL-1β in the culture medium was measured by ELISA. **(C)** Comparison of human and mouse sequences around the Ets motif. Data are presented as the mean + S.D. (*n* = 3). **(A)** *p < 0.05, two-tailed student’s *t*-test analysis. **(D)** *p < 0.05, Tukey–Kramer test.

A previous study using *Irf8*^-/-^ mice demonstrated that IRF8 is not involved in activation of the mNLRP3 inflammasome in macrophages (43), which seemed to be inconsistent with the results of the current study. To clarify the involvement of IRF8 in expression of NLRP3 in mouse macrophages, we introduced *Spi1* siRNA and *Irf8* siRNA into BMDMs and measured the expression of mNLRP3. As shown in **Figures 6A, B**, the levels of mNLRP3 mRNA and protein were significantly decreased in BMDMs in which PU.1 siRNA had been introduced but not in cells in which IRF8 siRNA had been introduced. Although the similarity between the nucleotide sequences of the mouse and human NLRP3 promoters is not high and the EICE identified in human *NLRP3* is not conserved in mice, a sequence similar to that of the Ets identified in the human gene was located around the transcription start site of mouse *Nlrp3* (**Figure 6C**). Moreover, the mRNA levels of *Pycard*, *Casp1*, and *Gsdmd* were significantly decreased by PU.1 knockdown but not by IRF8 knockdown (**Figure 6A**). Consistent with these changes, IL-1β secretion in response to LPS and ATP stimulation was markedly suppressed by PU.1 knockdown (**Figure 6D**). These results suggest that PU.1 and IRF8 cooperatively regulate *hNLRP3* expression, but only PU.1 affect the expression of *mNlrp3*.

## Discussion

In this study, we investigated the molecular mechanisms underlying macrophage-specific expression of human *NLRP3*. Within the distal promoter that is most active in monocytes and macrophages, we identified two *cis*-elements: an EICE at -309/-300 that binds a PU.1/IRF8 heterodimer and an Ets motif at +5/+8 that binds PU.1. Silencing of PU.1 and/or IRF8 by siRNA reduced the expression of hNLRP3. Accordingly, activation of the NLRP3 inflammasome was significantly disrupted by knockdown of PU.1 and/or IRF8 in human macrophages.

Although the expression levels of molecules related to the NLRP3 inflammasome were decreased by IRF8 knockdown to a degree similar as that imposed by PU.1 knockdown (**Figures 5A, D, E**), the suppressive effect of PU.1 knockdown on the release of hIL-1β protein was markedly higher than that imposed by IRF8 knockdown (**Figure 5F**). Production of hIL-1β in steady state THP-1 cells without stimulation (CTRL in **Figure 5F**) tended to be reduced when THP-1 cells were transfected with *SPI1* siRNA, whereas *IRF8* siRNA did not exhibit such an effect. Therefore, we hypothesized that this difference occurred because of the additional involvement of PU.1 in the transcription of *IL1B*, as Kominato *et al*. reported that PU.1 induces monocyte-specific *hIL1B* transcription by binding to the two Ets sites within the *IL1B* promoter (44). Indeed, we measured *IL1B* mRNA levels and found that the expression was significantly reduced by not only PU.1 knockdown but also IRF8 knockdown (**Supplementary Figure 2D**). Thus, PU.1 and IRF8 play two critical roles in IL-1β protein secretion: one is enhancing the conversion from precursor to biologically mature form by activating the NLRP3 inflammasome followed by Caspase-1 cleavage; the other is inducing the transcription of *IL1B* as a direct transactivator. Whereas maturation of Caspase-1 was completely inhibited by PU.1 knockdown, the stimulation-induced cleavage was somewhat promoted by IRF8 knockdown (**Figure 5E**). This difference is considered to have determined the amount of mature hIL-1β produced when THP-1 cells were transfected with either SPI1 siRNA or IRF8 siRNA and stimulated with LPS and nigericin. Further studies of the process of Caspase-1 cleavage are needed to understand the difference in hIL-1β secretion between PU.1 and IRF8 knockdown.

Although PU.1 knockdown significantly decreased NLRP3 expression in both human and mouse macrophages, the suppressive effect of IRF8 knockdown on NLRP3 expression was only observed in human cells (**Figures 6A, B**). Indeed, the EICE, which we identified as a PU.1/IRF8 heterodimer binding site within the *hNLRP3* promoter, does not seem to be conserved in the *mNlrp3* promoter, whereas the Ets site that can bind PU.1 is well-conserved. A previous study showed that activation of the NLRP3 inflammasome in *Irf8*^-/-^ BMDMs is unchanged from that in *Irf8*^+/+^ BMDMs (43). We cannot exclude the possibility that IRF4, which showed increased expression in IRF8 siRNA-introduced BMDMs (**Figure 6A**), compensated for the loss of IRF8. At least at the EICE, IRF8 appears to be required for expression of *hNLRP3* in human macrophages but is not engaged in *mNlrp3* transcription in mouse macrophages. It has also been recently reported that IRF8 suppresses *mNlrp3* expression in cDC1 and that IRF8 overexpression in mouse BMDMs causes a reduction in *mNlrp3* expression (33). These observations suggest that IRF8 plays opposite roles in the expression of NLRP3 in human and mouse macrophages.

Notably, *Irf8*^-/-^ mice showed defects in activation of the NLRC4 inflammasome because of downregulated expression of *mNlrc4* (43). Here, we demonstrated that *mNlrc4* mRNA expression was not decreased by IRF8 knockdown in BMDMs. This difference may be caused by knockout and knockdown of IRF8. In contrast, *hNLRC4/mNlrc4* mRNA expression was consistently downregulated by PU.1 knockdown in human and mouse macrophages (**Supplementary Figures 2A–C** and **3**). As PU.1 knockdown also decreased the level of *hNAIP/mNaips* mRNA in THP-1 cells, human macrophages, and BMDMs (**Supplementary Figures 2A, C** and **3**), it is possible that PU.1 modulates activation of the NLRC4 inflammasome in addition to the NLRP3 inflammasome.

Through reporter assays and EMSA, we demonstrated that three *cis*-elements are located within the *hNLRP3* promoter, one of which is recognized by the PU.1-IRF8 heterodimer and another by the PU.1 monomer. We could not identify the transcription factor(s) that binds to the region around -95/-61 of the *hNLRP3* promoter to contributes to its transcription. Given that deletion of this element significantly reduced reporter activity, this unknown transcription factor(s) may also play an important role in monocyte lineage-specific expression of NLRP3. Further studies are needed to fully understand the mechanisms by which *NLRP3* expression is regulated. Overall, we demonstrated that PU.1 and IRF8 contribute to monocyte/macrophage-specific expression of *hNLRP3*. They positively regulate *hNLRP3* transcription by forming a heterodimer on the -309/-300 EICE, which does not exist in the *mNlrp3* promoter. PU.1 regulates *hNLRP3* transcription by binding to the +5/+8 Ets site as a monomer. This mechanism is well-conserved between human and mouse macrophages.

## Data Availability Statement

The original contributions presented in the study are included in the article/**Supplementary Material**. Further inquiries can be directed to the corresponding author.

## Ethics Statement

The studies involving human participants were reviewed and approved by The Institutional Review Board of Tokyo University of Science. The patients/participants provided their written informed consent to participate in this study.

## Author Contributions

TY designed research, performed experiments, analyzed data, and wrote the paper. MY and SA performed experiments and analyzed data. MH and KY performed experiments. KU and KK provided experimental tools. CN designed research and wrote the paper. All authors contributed to the article and approved the submitted version.

## Funding

This work was supported by the Grant-in-Aid for Scientific Research (B) (CN; 20H02939), Grant-in-Aid for Scientific Research (C) (TY; 19K05884), Grant-in-Aid for Young Scientists (B) (TY; 17K15275), the MEXT-Supported Program for the Strategic Research Foundation at Private Universities (Translational Research Center, Tokyo University of Science), Tokyo University of Science Grant for President’s Research Promotion (CN), the Tojuro Iijima Foundation for Food Science and Technology (CN and TY), and the Takeda Science Foundation (CN). TY was supported by Research Fellowships of the Japanese Society for the Promotion of Science for Young Scientists (JSPS Research Fellowships for Young Scientists #10792).

## Conflict of Interest

The authors declare that the research was conducted in the absence of any commercial or financial relationships that could be construed as a potential conflict of interest.
