# Supplementary material for: PU.1 and IRF8 Modulate Activation of NLRP3 Inflammasome via Regulating Its Expression in Human Macrophages
Source: Front Immunol. 2021 Apr 7;12:649572. doi: 10.3389/fimmu.2021.649572 (PMC8058198; doi:10.3389/fimmu.2021.649572)
Supplement: Supplementary file 1 [file DataSheet_1.docx]

**Supplementary Table 1**

TaqMan gene expression assays for qPCR

| **Gene** | **Cat. No.** |
| --- | --- |
| *hSPI1* | HS02786711 |
| *hIRF8* | HS00175238 |
| *hGAPDH* | 432631E |
| *mSpi1* | Mm00488142_m1 |
| *Gapdh* | 4352329E |

Synthesized oligonucleotide primers for qPCR

| **Gene** | **Direction** | **Sequences** |
| --- | --- | --- |
| *hNLRP3* | Forward | GCAAGCTTCAGGTGTTGGAATT |
|  | Reverse | AAGATCCCAGCAGCAGTGTGA |
| *hPYCARD* | Forward | ATCCAGGCCCCTCCTCAGT |
|  | Reverse | GTTTGTGACCCTCGCGATAAG |
| *hCASP-1* | Forward | AAAAAATCTCACTGCTTCGGACAT |
|  | Reverse | TCTGGGCGGTGTGCAAA |
| *hGSDMD* | Forward | TTGACTCTGACTTGGACGTCCTT |
|  | Reverse | CGCGGGTGGCTGGAA |
| *hNAIP* | Forward | AGCTTATTGCAATGACAGCATCTT |
|  | Reverse | TGCAACTCCCACAGCTGATTC |
| *hNLRC4* | Forward | TGTGTTATAGAAAGGTGGGAAGCTT |
|  | Reverse | TTGAATAAGGGCTCGGCTATTG |
| *hAIM2* | Forward | AGCAACGTGCTGCACCAA |
|  | Reverse | GCTGGGCCACCATCTGTT |
| *hIL1B* | Forward | TCAGCCAATCTTCATTGCTCAA |
|  | Reverse | TGGCGAGCTCAGGTACTTCTG |
| *mIrf8* | Forward | CCGGATATGCCGCCTATG |
|  | Reverse | GCTGATGACCATCTGGGAGAA |
| *mNlrp3* | Forward | GCCTCCTGCAGAGCCTACAG |
|  | Reverse | CTTCTAAGGCACGTTTTGTTTCAC |
| *mPycard* | Forward | GCCAGTGTCCCTGCTCAGA |
|  | Reverse | GACCCTGGCAATGAGTGCTT |
| *mCasp-1* | Forward | AAGATTTTATTGCTTTCTGCTCTTCA |
|  | Reverse | TGAGCCCCTGACAGGATGTC |
| *mGsdmd* | Forward | CTCCTCAGGTGACAGAAAAGCA |
|  | Reverse | GCGCAGCAAGCACATTGA |
| *mNaip1* | Forward | GTTACCAGATCAGCTCTTCCATAACTT |
|  | Reverse | CATCCGGTTTGCCATCTAGTC |
| *mNaip2* | Forward | TGACAGCGTCTTCGCTAATGA |
|  | Reverse | GCCCCGGGTGATTCGT |
| *mNaip5* | Forward | CTTCCAGAAGCCACGGAAAC |
|  | Reverse | GAATGAACTGCTGCTGCATCA |
| *mNaip6* | Forward | AGAGCCAATATGCACTTCCAGAA |
|  | Reverse | GCTGCATCATCATGGTTGCT |
| *mNlrc4* | Forward | GGGAATGAAGCTCTACAGGAACTG |
|  | Reverse | CAATGTAGTGAGCTCTCCCAGAAC |

**Supplementary Table 2.** Sequence of primers used in construction of reporter vectors.

| Region | Site | Direction | Sequence |
| --- | --- | --- | --- |
| Distal | -600 | Forward | GGCAGGTACCCCGTCTCATGAGCTGCAGAG |
| Distal | -417 | Forward | GGCAGGTACCGCCTTCCAGCCATCTTGCTG |
| Distal | -226 | Forward | GGCAGGTACCCAGCCCCATCTCATACTGGC |
| Distal | -130 | Forward | GGCAGGTACCCAACACTGTTGATCCCATTTGG |
| Distal | -95 | Forward | GGCAGGTACCGTGATTAACCATTAGTCTCTCTG |
| Distal | +31 | Reverse | GTTACTCGAGCCAGCCTCAGGAACAGCTAG |
| Proximal | -673 | Forward | GCATGCTAGCGTGCCTAGCCTGTGGAAAGC |
| Proximal | +73 | Reverse | GCTAGATATCTGTCCCGTTGATTACGGGGC |

| Mutation site | Direction | Sequence |
| --- | --- | --- |
| EICE | Forward | AGTTGTCGACCCCTCACTCTCAGTGGAG |
|  | Reverse | GAGGGGTCGACAACTAAGGACATGCCATG |
| Ets | Forward | TGCAGTCGACCTCTAGCTGTTCCTGAGG |
|  | Reverse | TAGAGGTCGACTGCAATGAATTTATAGCA |
