# Supplementary material for: PU.1 and IRF8 Modulate Activation of NLRP3 Inflammasome via Regulating Its Expression in Human Macrophages
Source: Front Immunol. 2021 Apr 7;12:649572. doi: 10.3389/fimmu.2021.649572 (PMC8058198; doi:10.3389/fimmu.2021.649572)
Supplement: Supplementary file 2 [file DataSheet_2.docx]

**Supplemental Figure Legends**

**Sup. Fig. 1 Confirmation of the effects of another siRNA on expression of NLRP3.**

THP-1 cells were transfected with *SPI1* siRNA (HSS186058) (siPU.1-1), *IRF8* siRNA (HSS105169) (siIRF8-1), or negative control siRNA (siNega) and incubated for 48 hours. Relative mRNA expression was determined using qPCR and normalized to expression of Gapdh mRNA. Data are presented as the mean + S.D. (n = 3). **p* < 0.05, two-tailed student’s t-test analysis.

**Sup. Fig. 2 Effects of PU.1 and/or IRF8 knockdown on expression of the other NLRs family members.**

(A-D) Relative mRNA expression in THP-1 (A,D), U937 (B), or human macrophages (C) was determined by performing qPCR with cDNA same as Fig. 5A-C and normalized to expression of Gapdh mRNA. Data are presented as the mean + S.D. (n = 3). **p* < 0.05, two-tailed student’s t-test analysis.

**Sup. Fig. 3 Effects of PU.1 and/or IRF8 knockdown on expression of *Naips* and *Nlrc4*.**

Relative mRNA expression was determined by performing qPCR with cDNA same as Fig. 6A and normalized to expression of Gapdh mRNA. Data are presented as the mean + S.D. (n = 3). **p* < 0.05, two-tailed student’s t-test analysis.
