# Supplementary figures and images for: PU.1 and IRF8 Modulate Activation of NLRP3 Inflammasome via Regulating Its Expression in Human Macrophages
Source: Front Immunol. 2021 Apr 7;12:649572. doi: 10.3389/fimmu.2021.649572 (PMC8058198; doi:10.3389/fimmu.2021.649572)

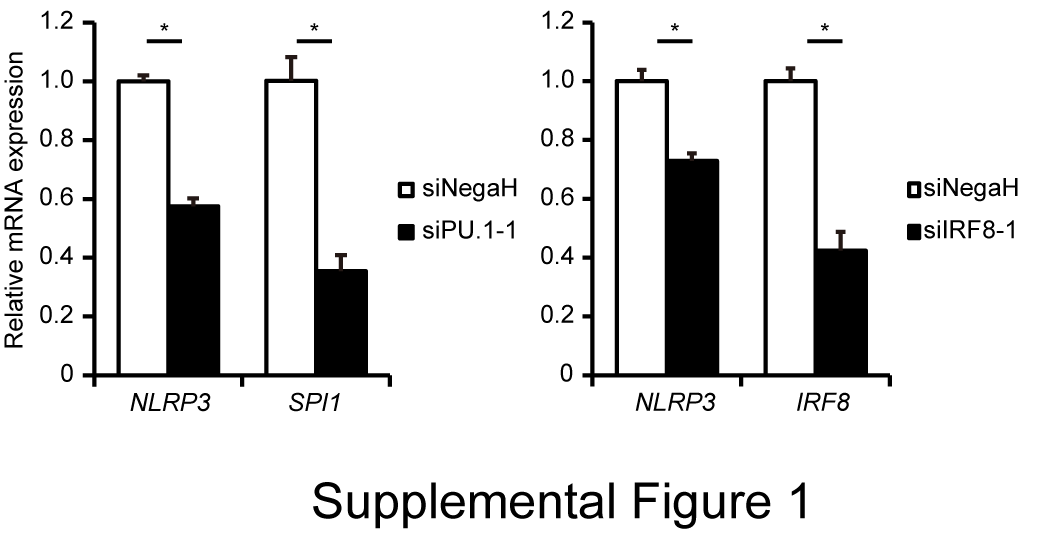

Supplement: Supplementary file 3 [file Image_1.tif]

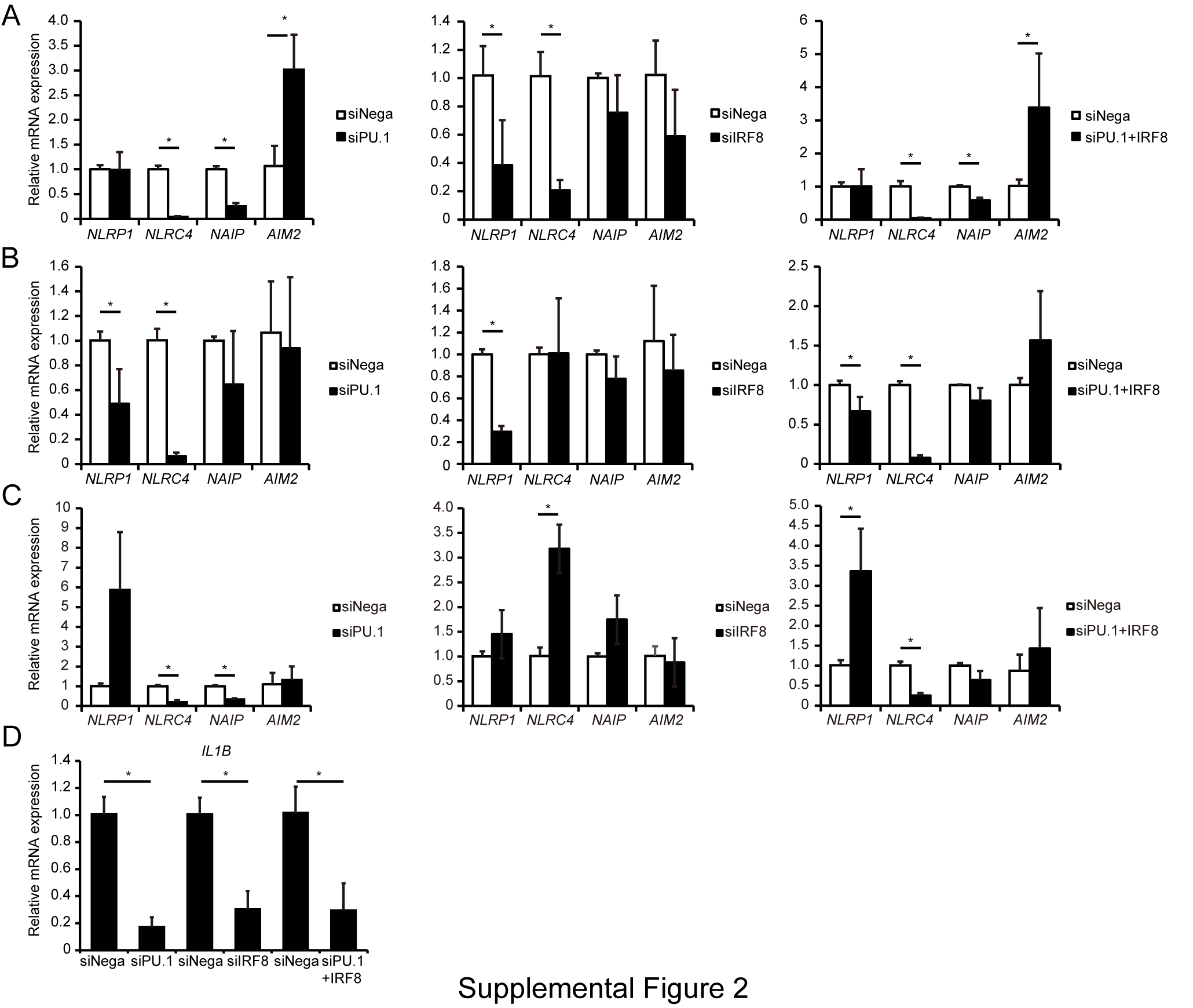

Supplement: Supplementary file 4 [file Image_2.tif]

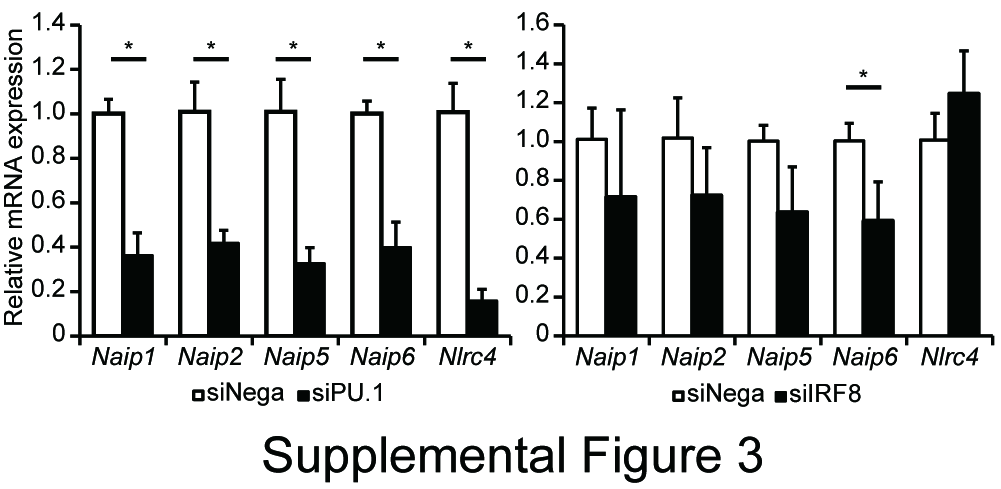

Supplement: Supplementary file 5 [file Image_3.tif]
